# Effects of Home-Based Electrical Stimulation on Plasma Cytokines Profile, Redox Biomarkers, and Metalloproteinases in the Heart Failure with Reduced Ejection Fraction: A Randomized Trial

**DOI:** 10.3390/jcdd9120463

**Published:** 2022-12-15

**Authors:** Marianne Lucena da Silva, Ivo Vieira de Sousa Neto, Alexandra C. G. B. de Lima, Fabrício Barin, Otávio de Toledo Nóbrega, Rita de Cássia Marqueti, Graziella F. B. Cipriano, João Luiz Quagliotti Durigan, Eduardo Antônio Ferreira, Martim Bottaro, Ross Arena, Larry P. Cahalin, José Alberto Neder, Gerson Cipriano Junior

**Affiliations:** 1 Rehabilitation Sciences and Health Sciences and Technologies Ph.D. Program, University of Brasilia (UnB), Campus Universitário, s/n, Centro Metropolitano, Brasilia 72220-275, DF, Brazil; 2 Health Sciences Academic Unit, Federal University of Jataí, Jataí 75801-615, GO, Brazil; 3 Department of Medicine, University of Brasilia (UnB), Campus Universitário Darcy Ribeiro, Asa Norte, Brasilia 70910-900, DF, Brazil; 4 Department of Pharmacy, University of Brasilia (UnB), Campus Universitário, s/n, Centro Metropolitano, Brasilia 72220-275, DF, Brazil; 5 Department of Physical Education, University of Brasilia (UnB), Campus Universitário Darcy Ribeiro, Asa Norte, Brasilia 70910-900, DF, Brazil; 6 Department of Physical Therapy, University of Illinois, 1919 W Taylor St., Chicago, IL 60612, USA; 7 Department of Physical Therapy, Leonard M. Miller School of Medicine, University of Miami, 5915 Ponce de Leon Blvd., 5th Floor, Coral Gables, FL 33101, USA; 8 Department of Medicine, School of Medicine at the Queen’s University, Queen’s University & Kingston General Hospital, Etherington Hall, Rooms 3032-3043, 94 Stuart Street, Kingston, ON K7L 3N6, Canada

**Keywords:** matrix metalloproteinases, oxidative stress, low-frequency electrical stimulation

## Abstract

Background: Low-frequency electrical stimulation (LFES) is an adjuvant method for heart failure (HF) patients with restrictions to start an exercise. However, the impact on molecular changes in circulating is unknown. We investigated the effects of 10 weeks of home-based LFES on plasma cytokines profile, redox biomarkers, metalloproteinases (MMPs) activity, and exercise performance in HF patients. Methods: Twenty-four HF patients (52.45 ± 9.15 years) with reduced ejection fraction (HFrEF) (EF < 40%), were randomly assigned to a home-based LFES or sham protocol. Plasma cytokines profile was assessed through interleukins, interferon-gamma, and tumor necrosis factor levels. Oxidative stress was evaluated through ferric reducing antioxidant power, thiobarbituric acid-reactive substances, and inducible nitric oxide synthase. The MMPs activity were analyzed by zymography. Cardiorespiratory capacity and muscle strength were evaluated by cardiopulmonary test and isokinetic. Results: LFES was able to increase the active-MMP2 activity post compared to pre-training (0.057 to 0.163, *p* = 0.0001), while it decreased the active-MMP9 (0.135 to 0.093, *p* = 0.02). However, it did not elicit changes in cytokines, redox biomarkers, or exercise performance (*p* > 0.05). Conclusion: LFES protocol is a promising intervention to modulate MMPs activity in HFrEF patients, although with limited functional effects. These preliminary responses may help the muscle to adapt to future mechanical demands dynamically.

## 1. Introduction

Heart failure with reduced ejection fraction (HFrEF) is a complex multisystem syndrome characterized by cardiac dysfunction that impairs cardiac output, leading to inadequate tissue perfusion [1]. This reduced tissue perfusion may trigger central and peripheral pathophysiological mechanisms [2], including vascular dysfunctions, abnormal skeletal muscle remodeling, metabolic capacity impairments, and loss of muscular fatigue resistance. These harmful responses may accelerate skeletal muscle protein degradation, reducing muscle strength and functional capacity [3], contributing to weakness and unfavorable prognosis [4].

A prolonged inflammatory response has been recognized as a critical contributor to functional disability in HF patients [5]. The cytokines are small secreted molecules that contribute significantly to HF pathophysiology out of the complex interplay between immune cells and inflammatory mediators [6]. It is well recognized that INF-γ, IL-2, IL-6, and TNF-α play crucial roles in the inflammatory cascade [7], accounting for systemic and muscle inflammation, possibly by pro-inducing matrix metalloproteinases (MMPs) production [5]. The MMPs are endopeptidases responsible for degrading structural extracellular matrix (ECM) components. Muscle ECM remodeling is accompanied by enhanced MMPs activity and may be represented by changes in their circulating concentrations. MMP-2 and MMP-9 are two important enzymes positively correlated with myocardial interstitial fibrosis and muscle abnormalities during HF development [8]. More recently, it was observed that oxidative stress also modulates MMPs activity, which in turn augments maladaptive ECM remodeling [9].

Evidence suggests mutual crosstalk between oxidative stress and inflammatory state regardless of HF comorbidities [10,11]. It has been demonstrated that oxidative markers of lipid damage (i.e., TBARS) and iNOS expression correlated positively with the severity of cardiac dysfunction and expression of proinflammatory cytokines through NF-κB pathways [12]. Meanwhile, ferric reducing antioxidant power (FRAP) and anti-inflammatory cytokines (IL-4 and IL-10) are attenuated in the HF condition [13]. Consequently, individuals are more susceptible to redox homeostasis challenges by having higher ROS levels and decreased antioxidant defense [14]. This redox imbalance might impair cell membrane integrity, DNA damage, and genomic instability, culminating in the apoptosis process in different tissues [15]. Therewith, it would be advantageous to identify inexpensive, low-risk, and accessible interventions that promote benefits in inflammatory, redox, and protein-change related biomarker state, improving health-related outcomes in HF patients with lower exercise capacity.

Low-frequency electrical stimulation (LFES), a frequency modality of neuromuscular electrical stimulation (NMES), has emerged as an exercise adjuvant method and an alternative preliminary method for severe HF with difficulty adhering to a regular exercise program [16]. The electrical stimulus emitted through motoneurons promotes axons depolarization and, consequently, involuntary contraction of the muscle fibers [17]. A meta-analysis on LFES in HF patients demonstrated improvements in peak oxygen consumption (VO_2_), VO_2_ at the ventilatory threshold, peak heart rate (HR), 6 min walk test (6MWT) distance, muscle strength, and flow-mediated dilatation (FMD) [18]. However, its response to peak oxygen consumption (VO_2_) seems to not be universal [19] and possibly only applied to highly severe patients [20]. Regarding molecular effectors, Karavidas et al. [21] observed downregulation of serum TNF-α and intercellular adhesion molecules (sICAM-1 and sVCAM-1) after a 6-week LFES in HF. However, the authors analyzed only some cytokines (IL-6, IL-10, and TNF-α) and adhesion markers, restricting physiological and molecular interpretations. The lack of cardiorespiratory fitness or muscle strength assessments and key players involved in the ECM environments and redox-sensitive signaling pathways might limit our understanding of cellular adaptation in response to LFES intervention.

Currently, serum molecular changes in HF patients contribute to peripheral vascular endothelial function and muscle remodeling after LFES has not been determined. The effects of LFES on redox biomarkers, cytokines, and MMPs in the blood circulation may contribute to new insights into training adaptations. This information would be valuable to elucidate possible protective mechanisms that are regulated by controlled intervention. Non-invasive biomarkers might undoubtedly improve our understanding of disease progression from a chronic perspective. Furthermore, no study has addressed the influence of LFES on maximum voluntary isometric contraction in HF patients. Therefore, this study aimed to investigate the effect of 10 week-LFES on plasma cytokines profile, redox biomarkers, MMP-2-9 activity, and exercise performance in HF patients. We hypothesized that LFES improves cardiorespiratory fitness and muscle strength and inhibits inflammatory activity, besides restores MMPs activity and general redox status, thus reducing plasma oxidative stress markers.

## 2. Materials and Methods

### 2.1. Study Design

A prospective, randomized, controlled trial was conducted in patients with HF with reduced ejection HFrEF (left ventricular ejection fraction < 40%) recruited in a Public Cardiology Hospital of Brasilia, Brazil, from June 2014 to July 2015. This was a 2-parallel group randomized clinical trial with a 1:1 intervention allocation. All procedures were approved by the Institutional Review Board (IRB) of the Cardiology Institute of Distrito Federal (ICDF protocol number 089/2011) in accordance with the Declaration of Helsinki, and all participants signed informed consent prior to study initiation. The trial was registered on Clinical Trial Registry (Protocol number NCT01695421) on 28 September 2012. Consolidated Standards of Reporting Trials and Statement for Randomized Trials of Nonpharmacologic Treatments (CONSORT) [22] were followed to conduct the present study.

### 2.2. Randomization and Allocation Concealment

A computer-generated randomization list was arranged using a computer-generated randomization schedule that distributed the participants into the sham or LFES group. One researcher (MLS) prepared sealed, opaque, and numbered envelopes. When each patient was enrolled in the study, the investigator opened the group’s smallest item number envelope.

### 2.3. Blinding

A blinded researcher (ASM) completed all functional assessments cardiopulmonary exercise testing and isokinetic testing). Laboratory analysis (Inflammatory Biomarkers, Oxidative stress, and Vascular Tone Signaling) was performed by another blinded researcher (RDM and FB). For this, another researcher (GCJ) performed the programming of each NMES tested and placed a black cover on the neuromuscular electrical stimulation device panel to maintain the parameters blinded for the researcher (MLS) and the participants, except for the intensity current. The researcher (MLS) who applied the currents and the participants was blinded to treatment allocation.

### 2.4. Participants

Individuals between 18 and 60 years of age, with a previous history of symptomatic heart failure caused by left ventricular systolic dysfunction [23] and clinical stability with optimized medical management for the past three months were included. The exclusion criteria were unstable angina, recent myocardial infarction, cardiac surgery within the previous three months, pacemaker or automated implanted cardioverter defibrillator, orthopedic limitations, or ongoing infectious disease. Additionally, patients with a previous diagnosis of pulmonary disease and active smokers were not included.

### 2.5. Study Flow

Participants were evaluated at baseline for initial clinical assessment (medical history, physical examination, and echocardiogram) and the baseline and the end of protocol for all outcome blood sample measures for laboratory analysis (plasma cytokines profile, redox biomarkers, and metalloproteinases activity) and exercise cardiopulmonary exercise testing (CPET), and isokinetic testing.

### 2.6. Low-Frequency Electrical Stimulation and Sham Group

Quadriceps and calf muscles in both legs were simultaneously electrically stimulated by a portable device (BLD Stim3, San Diego, CA, USA), submitted to a 1 h/day LFES or sham protocol, five days/week for ten weeks at home, after initial familiarization. A biphasic pulsed current was used with frequency: 25 Hz, phase duration: 400 μs, ON time: 10 s (including a rise time of 1 s and a decay time of 1 s, and off time: 20 s). The current amplitude was gradually increased while participants reported their discomfort using a 0–10 numeric scale after each LFES train, where 0 represented no discomfort and 10 represented the maximal perceived discomfort. According to a previous study, the current amplitude was adjusted in each session to produce maximum visible muscle contraction without an intolerant perceived discomfort [21]. The sham group was submitted to the same equipment and time intervention (frequency and weeks duration), however, with 0 mA intensity. All volunteers were instructed only to turn the equipment on and off, and next, they were instructed that when turning on the equipment after positioning the electrodes, they would be exposed to a noticeable neuromuscular electrical stimulation.

### 2.7. Echocardiogram

Images were acquired using an HD11x echocardiogram (Philips, Bothell, WA, USA) with a 2.5–3.5 MHz transducer. The standard M-mode and 2-dimensional echocardiography and the Doppler blood flow measurements were performed following the American Society of Echocardiography guidelines [24]. Septal and posterior left ventricle (LV) wall thickness were obtained from the parasternal long-axis view, and the LV end-systolic volumes (LVESV) were obtained from 2-dimensional apical images. According to Simpson’s method, left ventricular ejection fraction was calculated from 2-dimensional apical images [25]. The following variables were obtained: (1) aortic diameter and left atrial diameter measured in millimeters (Ao, mm; LAD, mm); (2) left ventricle end-diastolic volume in milliliters (LVEDV, mL); and (3) LV ejection fraction (LVEF, %)

### 2.8. Cardiopulmonary Exercise Test

Each individual performed a cardiopulmonary exercise test (CPET) to maximum tolerance on a treadmill ergometer (GE Marquette Case T 2100 Stress System Treadmill, GE Healthcare, Palatine, IL, USA) using an individualized ramp protocol. The modified ramping protocol selected for testing consisted of approximately 0.7 METS/min per min increases in workload every 60 s. Stage 1 began at 27 m·min^−1^ and 0% grade. Stages increased by 13.5 m·min^−1^ and 2% grade per min. Velocity was limited to 54 m·min^−1^ and 20% grade. The aim was to achieve peak exercise in ≈8 to 12 min. All participants were monitored for at least 6 min in the recovery phase.

Ventilatory expired gas analysis was obtained using a metabolic cart (MedGraphics VO2000^®^—Medical Graphics Corp., St. Paul, MN, USA). The oxygen and carbon dioxide sensors were calibrated prior to each test. For VO_2_ peak reproducibility, the study demonstrated ICC values higher than 0.9 but with a CV of 14.2% for this measurement [26]. Monitoring consisted of continuous 12-lead electrocardiography obtained with the CASE system (CardioSoft Diagnostic System, GE Healthcare, Palatine, IL, USA), manual blood pressure measurements with an aneroid sphygmomanometer (Tycos, Welch Allyn, Skaneateles Falls, NY, USA), heart rate recordings via the electrocardiogram, and rating of perceived exertion using the RPE (Borg 1 to 10) scale, all of them on each stage of the protocol. Test termination criteria consisted of patient request, ventricular tachycardia, ≥2 mm of horizontal or down-sloping ST-segment depression, or a drop in systolic blood pressure ≥ 20 mm/Hg during exercise. A certified exercise physician conducted each test. Oxygen consumption (VO_2_, mL kg min), dioxide carbon (VCO_2_, L min), and minute ventilation (VE, L min) were collected continuously throughout the exercise test. VO_2_ peak was expressed as the highest 30 s average value obtained during the last stage of the exercise test. Peak respiratory exchange ratio (RER) was the highest 30 s averaged value during the last stage of the test. Ten seconds averaged VE and VCO_2_ data, from the initiation of exercise to peak, were input into spreadsheet software (Microsoft Excel, Microsoft Corp., Bellevue, WA, USA) to calculate the VE/VCO_2_ slope via least-squares linear regression (y = mx + b, m = slope) [27]. All participants were asked about the occurrence of angina and symptoms at each stage of the exercise protocol.

### 2.9. Isokinetic Testing

Peak torque (PT) evaluation was performed on a Biodex III isokinetic dynamometer system (Biodex Medical Inc., New York, NY, USA). Participants were positioned on a chair, with free and comfortable flexion and extension movement of the knee, where the extension was defined as 0° and flexion as 90°, using a flexion-extension movement amplitude of 80° (excursion from 90° to flexion up to 10°). The femur’s lateral epicondyle was used as the knee’s reference axis of rotation aligned to the rotation axis. The protocol consisted of three maximal isometric voluntary contractions (MVC), each MVC lasting 5 s with 2 min between the repetitions. The highest peak torque between the three repetitions was recorded as the maximal value. We used the equation proposed by Harbo et al. [28] to evaluate the baseline isokinetic strength in our population. Studies including a variety of participants generally observed an excellent level of reliability for isokinetic dynamometers under test–retest conditions with intra-class correlation (ICC) values higher than 0.85 and coefficient of variation (CV) lower than 8.7% for knee evaluation [29,30].

### 2.10. Blood Sample Collection

Participants fasted overnight before each study, keeping their morning medications as prescribed. Blood sample collections were performed between 9 a.m. to 12 p.m. To measure serum redox biomarkers, cytokines, and metalloproteinases, blood samples (5 mL) were drawn from the antecubital vein 24 h after the end of a training period and posteriorly placed in BD Vacutainer^®^ Fluoret/EDTA tubes (Becton Dickinson, Franklin Lakes, NJ, USA). Plasma was separated immediately after collection, centrifuged at 2500 rpm for 15 min, and posteriorly stored at −80 °C for analysis.

### 2.11. Plasma Cytokines Profile

Plasma cytokines concentrations were assessed by a multiplexed bead-based immunoassay using one set of the Human Inflammatory Cytometric Bead Array (CBA) kit manufactured by the BD Bioscience SA (San Diego, CA, USA) and used according to the manufacturer protocols, which allowed six different measurements for circulating mediators, as follows: interleukin (IL) −2, −4, −6, −10, interferon-gamma (INF-γ) and tumor necrosis factor (TNF-α). The lyophilized cytokine standards and the plasma samples were processed, and the results were acquired using the BD FACSCalibur flow cytometer, FL4 channel (BD Bioscience SA, San Diego, CA, USA). Three hundred events were acquired for each cytokine bead used. Data were analyzed using the FCAP software, version 3.0 (BD Bioscience SA, San Diego, CA, USA). The concentration of cytokines was calculated by interpolation in the corresponding standard curve. Whenever both kits assessed a given cytokine, the mean value obtained was considered. All standards and samples were measured in duplicate. The overall intra- and inter-assay coefficients of variation for cytokines were within 2 to 10%

### 2.12. Redox Biomarkers

The plasmatic antioxidant capacity, lipid peroxidation, and vascular tone signaling were determined through ferric reduced antioxidant power (FRAP), the thiobarbituric acid reactive substances (TBARS), and induction of nitric oxide synthase (iNOS), respectively. The FRAP was determined by the capacity of the antioxidants to reduce Fe^+3^ to Fe^+2^ that is chelated by TPTZ (2,4,6-tris(2-pyridyl)-s-triazine) and form the complex Fe^+2^ TPTZ [31]. The quantification of lipid peroxidation products was determined by a commercial assay kit (Cayman Chemical, Houston, TX, USA). The formation of substances reacting to thiobarbituric acid was analyzed, and so too the final products of lipid peroxidation (lipid peroxides, malondialdehyde, and aldehydes) reacting with 2-thiobarbituric acid (TBA) to form Schiff bases. The amount of lipid peroxidation was reported as micromolar malondialdehyde equivalents [32]. The iNOS concentration was assessed by using a commercial assay EIA kit (cat no. EH0556; FineTest, Wuhan, China). This assay was based on sandwich enzyme-linked immune-sorbent assay technology and carried out according to the manufacturer’s instructions. Samples and standards were read at a wavelength of 420–450 nm. All the assays were measured using UV-visible spectroscopy with a microplate reader (SpectraMax^®^ M3 Multi-Monochrome, Orleans Drive, Sunnyvale, CA, USA) and calculated by analysis software (SoftMax^®^ Pro Data Acquisition, Version 7.0 June 2016, San Jose, CA, USA). These measurements concurred with the determination of 1.0 mL of plasma. All measurements were performed in duplicate to guarantee reliability. All redox state markers intra- and inter-assay CVs were within 2 to 15%.

### 2.13. Matrix Metalloproteinases Activity

The supernatant from plasma from previous analyses was used for the zymography technique. Biological replicates samples of patients containing 0.5 uL of plasma (normalized for the total amount of protein included) were added to 0.5 uL of SDS (8%) (v:v). Subsequently, they were placed in a vortex, and 10 uL of sample buffer without β-mercaptoethanol (reducing agent), containing SDS (20%) was added. Samples were resolved by electrophoresis in sodium dodecyl sulfate-polyacrylamide gel electrophoresis (SDS-PAGE-10%) and gelatin at a 1 mg/mL final concentration. Afterward, the gels were washed twice for 20 min in a 2.5% Triton X-100 to remove SDS solution and incubated at 37 °C for 20 h in a substrate buffer (50 mmol; L^−1^ Tris-HCl (pH 8.0), 5 mmol; L^−1^ CaCl_2_ and 0.02% NaN_3_). To ensure the accuracy of the analysis, the different samples from groups were prepared simultaneously using the same gels for zymography. Moreover, fresh buffers, voltage, time during electrophoresis, and gel staining background were carefully standardized to minimize variation between the MMPs’ activity. The gels were stained in Coomassie brilliant blue R-250 (Bio-Rad, Hercules, CA, USA) for 1 ½ h and detained with acetic acid, methanol, and water (2 ½ h). MMP-2 and MMP-9 activity were visualized as clear white bands (manifested as horizontal) against a blue background by densitometric scanning (Image Scanner III, Lab Scan 6.0, Geneva, Switzerland). The averages of the band intensities were measured using Image Master 2D Platinum 7.0 software (Björkgatan, Uppsala, Sweden) and conducted by a blinded researcher, attenuating possible bias related to this process. Pro and active isoform bands were identified via standard techniques using molecular weight criteria. The bands found in all groups were 95–64 kDa (Pro-MMP-9: 95 kDa; Active-MMP-9: 95 kDa; Pro-MMP-2: 72 kDa; Active-MMP-2: 64 kDa), as proposed by previous studies that evaluated MMP-2 and MMP-9 in the blood circulation [33,34].

### 2.14. Data Analysis and Sample Size Calculation

The normal distribution was verified using the Shapiro–Wilk test. According to this, data were expressed as mean ± SD. Two-Way ANOVA (time × group) with repeated measurements was used, followed by the Bonferroni post-hoc test. Data analysis and graphics design were performed using GraphPad Prism 8.3 (San Diego, CA, USA). Statistical significance was defined as *p* < 0.05 for all tests. An intention-to-treat analysis was performed for all randomized participants. Missing data were replaced using the expectation-maximization method. For cardiopulmonary Tests and Isometric Strength outcomes, 11 participants were assessed in LFES and 13 in the sham group. For cytokines and redox biomarker blood sample outcomes, 10 and 11 (LFES and sham) participants were assessed due to an error in biochemical analysis in 1 participant per group. For MMPs, 8 and 9 (LFES and sham, respectively) participants were assessed. The sample size was calculated using the TNF-α changes following the LFES protocol as a primary outcome. Using results from a previous pilot study with six participants, we determined that a 10% difference in TNF-α levels could be detected with 12 participants per group (α = 0.05, β = 0.20, and power = 80%).

## 3. Results

### 3.1. General Observations

**Figure 1:**
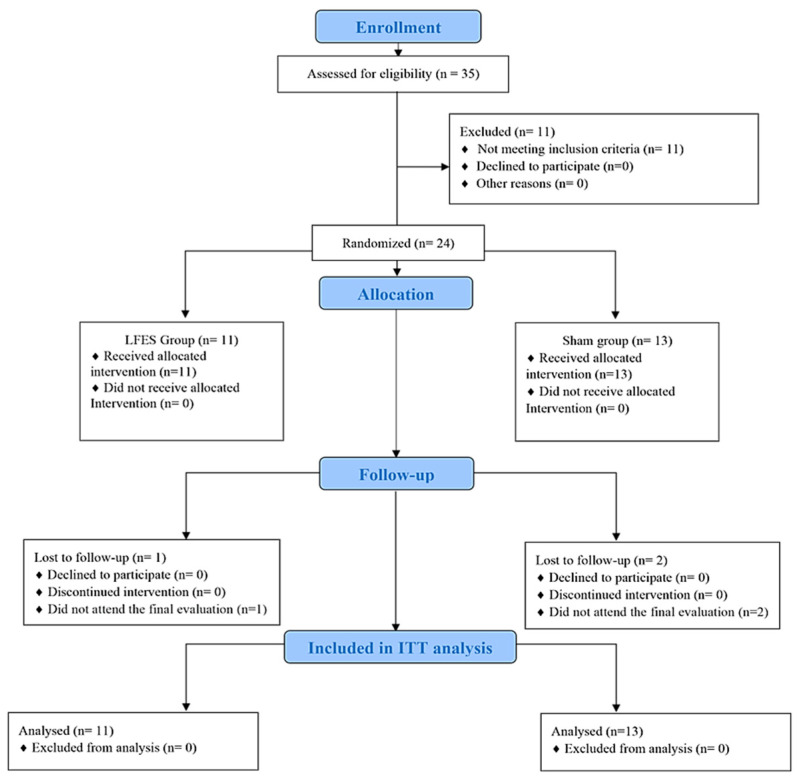
Flowchart of the randomized trial.

Thirty-five subjects were screened for the study. Eleven participants did not meet the inclusion criteria and were not included. Therefore, twenty-four participants (LFES, n = 11; sham, n = 13) were randomized. Of twenty-four participants, three (LFES, n = 1; sham, n = 2) were lost during the follow-up period due to hospitalization. (Figure 1). No cases of skin burn, or injury caused by FES, occurred.

Table 1 describes the clinical characteristics and demographics. There were no differences between groups in gender distribution, age, and HF etiology. Left ejection fraction was reduced in the LFES and sham groups. A random difference between New York Heart Association (NYHA) class after the randomization was observed. All patients received guideline-recommended therapy for HF, including digoxin, diuretics, angiotensin-converting enzyme inhibitors, and beta-blockers.

### 3.2. Effects Magnitude of LFES on Cardiopulmonary Exercise Test and Isokinetic Muscle Strength

There was no significant change in the cardiopulmonary exercise test variables and isometric muscle strength between the LFES and sham groups (*p* > 0.05; Table 2).

### 3.3. Effects Magnitude of LFES on Plasma Cytokines Profile and Redox Biomarkers

There were no significant changes between groups and time points for cytokines and redox biomarkers (*p* > 0.05; Table 3).

### 3.4. Effects Magnitude of LFES on Matrix Metalloproteinases Activity

**Figure 2:**
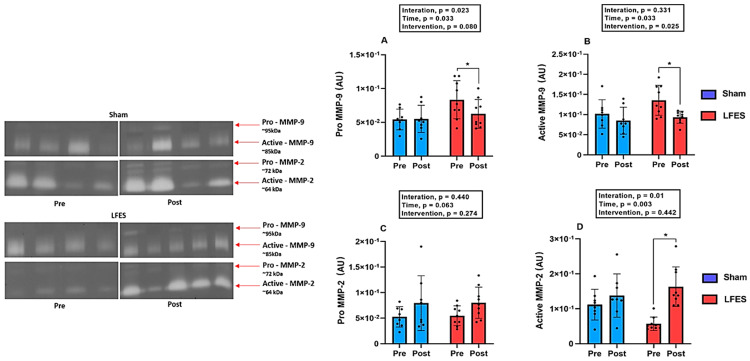
Low-frequency electrical stimulation (LFES) affects matrix metalloproteinase activity in heart failure with reduced ejection fraction (HFrEF). **Note***:* Data presented as mean (SD). Optical densitometry of zymographic bands of MMP-2 in arbitrary units (AU). (**A**) Pro MMP-9 activity; (**B**) Active MMP-9 activity; (**C**) Pro MMP-2 activity; (**D**) Active MMP-2 activity; Pre = baseline; Post = after 10 weeks; * *p* < 0.05.

Regarding Pro-MMP-9 activity, LFES showed lower values at post-training compared to pre-training (0.083 to 0.062, *p* = 0.002) (Figure 2A). Similarly, Active-MMP9 activity decreased in the LFES group after intervention (0.135 to 0.093, *p* = 0.02) (Figure 2B). There were no significant changes between groups and time points for Pro-MMP2 activity (*p* > 0.05; Figure 2C). However, we observed an increase of Active-MMP2 activity in the LFES group at post-training compared to pre-training (0.057 to 0.163, *p* = 0.0001) (Figure 2D).

## 4. Discussion

**Figure 3:**
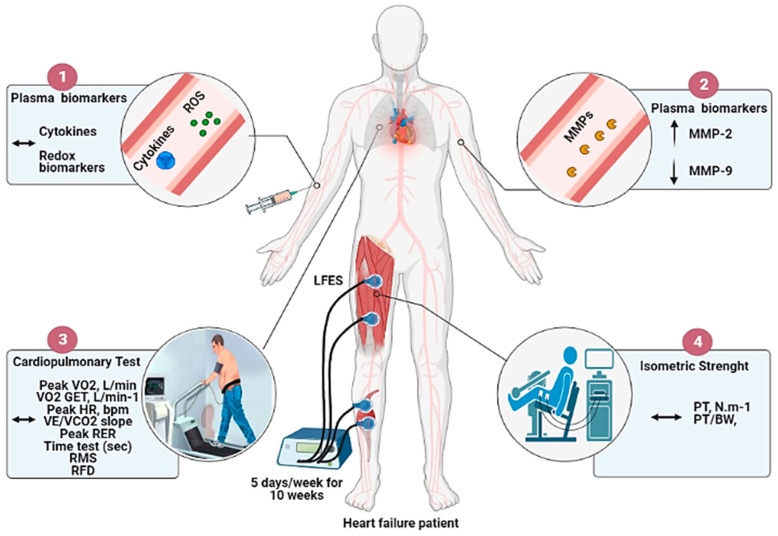
Overview of the effects of LFES on cytokines, redox biomarkers, metalloproteinases activity, cardiorespiratory fitness, and muscle strength in heart failure patients. Note: The 10 weeks of home-based low-frequency electrical stimulation (LFES) protocol: (**1**) Does not change plasma cytokines profile and redox biomarkers; (**2**) ↑ MMP-2 and ↓ MMP-9 activity; (**3**) Does not change cardiorespiratory fitness outcomes (**4**) Does not change isometric muscle strength.

The novel findings from this study are that a 10-week home-based LFES program in individuals with HFrEF downregulates serum MMP-9 activity, which can be helpful to control the inflammatory state. Most importantly, LFES upregulates MMP-2 activity in blood circulation, which can be attributed to ECM remodeling in response to muscle contraction. However, our entire initial hypothesis was not confirmed since the LFES by itself was not able to modify plasma cytokines profile, redox biomarkers, cardiorespiratory fitness, and muscle strength as a single intervention (Figure 3), suggesting limited effects on functional and key biomarkers. These results have important implications for HF patients who cannot enroll in an exercise-based cardiac rehabilitation program. Moreover, our findings may help discuss the minimum amount of LFES needed to obtain health benefits in the HF population without producing harmful effects caused by the onset of muscle fatigue (e.g., pain, fibrillation, and torque reduction).

A critical facet of HF condition is the diminished ability of the muscle to regenerate, repair, and remodel [35]. MMPs have been considered a potential biomarker due to their high sensitivity in signaling ECM changes through protease modulation. The proteolytic processing occurs due to the remodeling and degradation of the ECM from both physiologic and pathophysiologic stimuli on muscle tissue [33]. In skeletal muscle, MMPs play an essential role in myofiber functional integrity by regulating skeletal muscle cell migration [36,37], among which MMP-2 activity has been related to regeneration of new myofibers, probably due to type IV collagen degradation from the basement membrane during myoblast proliferation, migration, and fusion [36]. It is possible to suggest that LFES is an essential intervention capable of inducing significant effects on MMP-2 activity, which can be relevant to the muscle remodeling process, considered an important mechanism for tissue adaptation.

Prior studies presented similar findings on MMP-2 after prospective active exercise protocols in HF patients, including aerobic and resistance exercise programs [37]. Moreover, these findings related to MMP-2 activity after an LFES stimulation protocol were only previously described in animal studies [38,39]. Thus, this work represents an essential first step in understanding MMP-2 as a critical biochemical marker in clinical research, providing new insights into muscle development and regeneration following an LFES protocol. It may suggest using LFES as an alternative treatment to prepare the muscle for an exercise training program, given its demonstrated potential to modulate MMP2 activity [39,40]. These factors may help endow the LFES utilization in parallel to an exercise training program may enhance functional improvements. Future investigations should be conducted to explore these areas and solidify the clinical utility of LFES in patients with a chronic condition.

Meanwhile, MMP-9 may have distinct roles when compared to MMP-2 during clinical conditions. MMP-9 can be considered a key mediator of inflammation and, consequently, provide important inflammatory states [41]. It has been demonstrated that MMP-9 orchestrates the survival, evasion, and transmigration of multiple inflammatory cells (polymorphonuclear leukocytes, and monocytes) from the blood circulation to the site of inflammation in skeletal muscle by processing ECM components [34]. In the current study, LFES downregulated MMP-9 activity at post-training, implying a protective factor against the undesired effects of HF condition. Possibly, this adaptation is essential to alleviate musculoskeletal damage and the inflammatory microenvironment. Downregulation of MMP-9 activity in circulation is essential for HF patients since high levels of this enzyme are related to heart fibrosis, microvascular complications, peripheral arterial dysfunction, acute myocardial infarction, and unfavorable prognosis [42,43,44,45,46]. Thus, MMP-9 can be an important prognostic marker for the clinical course and evaluation of patients’ response to the exercise intervention.

There is evidence that inflammation and oxidative stress negatively affect muscle cell differentiation efficiency, which is a primary characteristic of chronic muscle disorders and muscle wasting [47]. Karavidas et al. [21] found an attenuation of the peripheral immune pathways through a TNF-α reduction following an LFES protocol in patients with HF. Even though we used similar LFES parameters for the protocol in the current study, 60 instead of 30 min a day, we did not demonstrate a beneficial effect on cytokines and balance redox, which might be influenced by training modality (home-based) and patient characteristics, including HF severity and baseline inflammatory activity. Moreover, Karavidas et al. [21] have also proposed that the release of vasorelaxant factors into the circulation, such as prostaglandins and nitric oxide, leads to an upregulation of anti-oxidative enzymes, attenuating nitric oxide degradation from free radicals. However, contrary to a possible mechanism suggested by Karavidas et al. [21], we did not find a relevant change of iNOS levels in our protocol, which indicates that other adjacent molecular pathways or post-translational regulation were involved in the local blood flow and vascular homeostasis control. In addition, whether LFES can modulate other protective antioxidant systems, intracellular signal transducer, and transcription factors involved in oxidative capacity remains a provocative hypothesis for further investigation. These mechanisms are essential for tissue microenvironment, metabolism homeostasis, and consequently cellular longevity.

Similar to Ennis et al. [19], cardiorespiratory fitness following the LFES protocol was not detected in our study, although observed in other studies [18]. These changes might be related to the fact that there is no singular pathway mediating neuromuscular and cardiovascular adaptations. Consequently, the pleiotropic effects of LFES in HF patients can also be modulated by multiple and complex responses at the molecular level. A delicate balance between numerous signaling pathways can improve functional improvements, and a minimum amount of combined voluntary exercise might be essential. Furthermore, the limited effects of our intervention on function and exercise performance can be partially explained by the profile of our sample since better results would be expected in more severely affected patients [20]. LFES protocol discrepancies on scientific literature (e.g., intensity graded from visual contraction to 30% of maximum contraction, frequency from 4 to 50 Hz, number of stimulated muscles from 1 to 4, stimulation duration from 30 to 240 min daily, from 6 to 10 weeks) may also explain various therapeutic responses [18]. There were no cases of bed-ridden patients with advanced HF who suffer from severe physical symptoms or complex comorbidities, suggesting that the participants could use dynamic exercise at higher intensities. Collectively, the complexity and profound variability in human function highlights the need to understand better the multidimensional array of interacting factors that determine the trajectory of effort tolerance and exercise capacity in HF conditions.

Additionally, although neuromuscular electrical stimulation has been recommended for HF patients, there is no clear evidence regarding the ideal parameters, including frequency (days per week), duration (session time), nor intensity, to produce functional gains. The responsiveness to LFES includes characteristics of the exercise regimen, dose, or effect. Presumably, different patients have distinct physiological adaptations, and, possibly, remodeling is not the same after the same neuromuscular electrical stimulation. Thus, future studies may investigate the variables modulated by training to potentiate the NMES effects and highlight several cornerstone mechanisms involved, besides effectiveness and optimal dosage. A more effective strategy would include manipulating different electrical stimulation currents, phase durations, intensity increment and discomfort, and NMES-efficiency in generating evoked-torque.

The contrast between our study and Dobsak et al. [48] could be related to several factors, including (1) different heart failure severity patients included (NYHA Class II-III vs. II-IV), (2) weekly electrical stimulation volume (5 vs. 7 h per week), (3) LFES application modality employed (self-prescription vs. laboratory-controlled manner). Furthermore, we used 20 Hz of Frequency to hamper muscle fatigue [49]; however, for muscle strength adaptation, the literature clearly states to use 100 Hz [49]. Nevertheless, there is poor information on whether this discrepancy in Frequency might trigger the mechanism of peripheral skeletal muscle adaptation and strength, as observed in healthy subjects.

Despite using evidence-based therapies, long-term morbidity and mortality remain unacceptably high among HF patients [50,51,52]. One of the essential aspects of the present research is to encourage HF patients to perform exercise training, as we identified that LFES protocol is a promising stimulus to modulate MMPs activity, which might have critical implications for prescription and health outcomes. In this fashion, to aid the successful transfer to exercise modalities, adherence, adhesion, unwillingness, and dropout reasons for an exercise program should be considered for HF patients. Although we did not perceive functional changes, LFES can be a potential tool for preparing skeletal muscle, supporting new exercise training routines, higher loads and exercise tolerance. Prior molecular signaling can be preliminary for beneficial adaptations in the tissue environment and potentially translate into improved clinical outcomes and decreased hospitalization risk.

Some limitations of the present study should be highlighted, such as the absence of enzymatic ROS scavengers, such as superoxide dismutase, glutathione peroxidase, and catalase, relevant to physiological status modulation in response to exercise, since they involve molecular processes mediating oxidative and non-oxidative. Another limitation to consider was the inability to analyze the body composition by dual-energy X-ray absorptiometry, which could have helped better to understand the beneficial effects of LFES on muscle mass. Moreover, post-LFES MMPs activity is the same as the post-sham, which may be related to pre-intervention biological variability inherent to HF condition; Although the study has utilized several recognized measures to control all pre-specified inclusion criteria (clinically stable heart failure patients with reduced ejection fraction phenotype and without acute cardiovascular conditions), underlying CV pathologies may have influenced the pre-intervention MMP values. However, considering the study was adequately randomized, we believe the post-intervention change provides a valid estimate of the treatment effect. Finally, despite reaching the expected sample number according to a priori power calculation, we acknowledge that this number may not be enough for all the study’s secondary variables. However, considering the effect magnitude observed for the main variables (plasma cytokines profile, redox biomarkers, MMP-2-9 activity, and exercise performance), the MMP activity also had sufficient power to demonstrate the effects of the LFES protocol. Regarding the other outcomes, we believe that only the outcome that would benefit from a higher number would be the Isometric Strength since a clinically important non-reduction trend was observed in the LFES group but not in the sham group.

In addition, skeletal muscle biopsy would be necessary to shape these conjectures and examine mechanisms at a cellular level and the changes in muscle fiber. However, serum cytokines, redox, and metalloproteinases biomarkers have been recognized as a less invasive and reliable measure to analyze possible muscle responses. Finally, few studies are available on semi-supervised protocol and patient characteristics, so further well-designed RCTs are needed to better understand the effects of LFES in a home-based program in HF patients.

## 5. Conclusions

The LFES protocol applied for 10 weeks in a home-based program in HFrEF patients downregulates MMP-9 activity while possibly stimulating muscle remodeling through an active MMP-2 activity increase. These responses may help the muscle to adapt to future mechanical demands dynamically. However, the LFES protocol did not modify plasma cytokines profile, redox biomarkers, cardiorespiratory fitness, or muscle strength, suggesting that other adjacent molecular pathways were involved in the LFES responsiveness. Our findings provide new insights into training adaptations for HF patients and non-invasive biomarkers to improve our understanding of disease progression from a chronic perspective.

## Figures and Tables

**Table 1:** Demographic characteristics and clinical history of HF patients.

Variables	LFES (n = 13)	Sham (n = 11)
**Demographics**		
Age, years	53.7 ± 9.8	51.2 ± 8.5
Height, cm	165.7 ± 10.4	166.7 ± 9.1
Weight, kg	76.7 ± 17	74.7 ± 18.8
**Gender, n (%)**		
Male/Female	9 (69.2)/4 (30.8)	8 (72.7)/3 (27.2)
**History clinical, n (%)**		
Obesity	2 (15.4)	2 (18.1)
Smokers	0	0
Diabetes mellitus	9 (69.2)	10 (90.9)
**HF etiology, n (%)**		
I-HF	7 (53.8)	6 (54.5)
C-HF	6 (46.2)	5 (45.4)
**NYHA, n (%)**		
II	3 (23.1)	3 (27.2)
III	3 (23.1)	4 (36.3)
IV	7 (46.2)	4 (36.3)
**Echocardiography**		
LA diameter, mm	48.7 ± 7.4	46.3 ± 4.4
Ejection fraction, %	29.1 ± 7.4	30 ± 5.5
Ao (mm)	34.3 ± 4.5	33.0 ± 4.0
RA (mm)	48.7 ± 7.3	46.8 ± 4.1
FDV (mL)	246.9 ± 98.1	232.9 ± 4.4
**Medications, n (%)**		
Diuretic	10 (76.9)	8 (72.7)
Spironolactone	10 (76.9)	9 (81.8)
ACEI	13 (84.6)	10 (90.9)
Digoxin	7 (53.8)	4 (36.3)
Beta-Blocker	11 (84.6)	11 (100)

**Note**: Data presented as mean (SD). Number and in percentage (%). LFES, low-frequency electrostimulation; I-HF, ischemic heart failure; C-HF, Chagas heart failure; LA, left atrium diameter; NYHA New York Heart Association; ACEI, angiotensin-converting enzyme inhibitors; Ao, Aorta; RA, Right Atrium; FDV, End Diastolic Volume.

**Table 2:** Cardiopulmonary capacity and muscle strength following LFES intervention.

					Within-Group Difference				Between-Group Difference			
					(10w vs. Baseline)				(Sham vs. LFES)			
	Sham		LFES		Sham		LFES		Baseline		10 Weeks	
Variables	Baseline	10 Weeks	Baseline	10 Weeks	Mean Difference	(95% CI)	Mean Difference	(95% CI)	Mean Difference	(95% CI)	Mean Difference	(95% CI)
**Isometric Strenght**	n = 13	n = 13	n = 11	n = 11								
PT. N m^−1^	168.0 ± 55.00	174.0 ± 59.00	171.0 ± 53.00	171.0 ± 53.00	−6.7	−17 to 4.0	−0.58	−12 to 11	−2.8	−55 to 50	3.3	−49 to 56
PT/BW. N m^−1^ Kg	212.0 ± 56.00	219.0 ± 51.00	227.0 ± 47.00	226.0 ± 43.00	−7	−26 to 12	1.1	−19 to 21	−14	−65 to 36	−6.3	−57 to 45
**Cardiopulmonary Test**	n = 13	n = 13	n = 11	n = 11								
Peak VO_2_. L/min	15.0 ± 4.60	16.0 ± 4.80	18.0 ± 4.00	19.0 ± 4.40	−0.21	−1.6 to 1.2	0.69	−0.82 to 2.2	−1.9	−4.8 to 0.93	−1	−3.9 to 1.8
VO_2 GET_. L/min^−1^	13.0 ± 2.90	13.0 ± 3.60	15.0 ± 2.80	14.0 ± 2.50	−0.015	−4.0 to 3.9	−2.5	−6.8 to 1.8	3.6	−4.1 to 11	1.1	−6.6 to 8.8
VE/VCO_2_ *slope*	37.0 ± 7.40	37.0 ± 6.80	33.0 ± 6.10	36.0 ± 11.00	−1.1	−2.5 to 0.31	−0.18	−1.7 to 1.3	−3.2	−7.5 to 1.0	−2.3	−6.6 to 2.0
Time test (s)	393.0 ± 133.00	412.0 ± 77.00	485.0 ± 120.00	425.0 ± 108.00	−27	−99 to 45	66	−10 to 142	−92	−201 to 17	0.76	−113 to 114

**Table 3:** Plasma cytokines profile and redox biomarkers following LFES intervention.

					Within-Group Difference				Between-Group Difference			
					(10w vs. Baseline)				(Sham vs. LFES)			
	Sham		LFES		Sham		LFES		Baseline		10 Weeks	
Variables	Baseline	10 Weeks	Baseline	10 Weeks	Mean Difference	(95% CI)	Mean Difference	(95% CI)	Mean Difference	(95% CI)	Mean Difference	(95% CI)
**Cytokines**	n = 12	n = 12	n = 10	n = 10								
IL-2 (pg/mL)	4.00 ± 0.35	4.40 ± 0.99	4.10 ± 0.70	4.20 ± 0.42	−0.42	−1.1 to 0.28	−0.098	−0.86 to 0.66	−0.16	−0.83 to 0.52	0.16	−0.51 to 0.83
IL-4 (pg/mL)	0.42 ± 0.29	0.69 ± 0.49	0.72 ± 0.63	0.54 ± 0.32	−0.27	−0.66 to 0.12	0.18	−0.25 to 0.61	−0.3	−0.75 to 0.15	0.15	−0.30 to 0.60
IL-6 (pg/mL)	6.80 ± 6.30	5.30 ± 4.40	5.20 ± 5.10	4.10 ± 2.80	1.5	−2.0 to 5.0	1.1	−2.7 to 4.9	1.5	−3.3 to 6.4	1.2	−3.7 to 6.0
IL-10 (pg/mL)	2.20 ± 1.60	2.00 ± 0.87	2.70 ± 2.00	2.90 ± 2.50	0.23	−1.7 to 2.2	−0.19	−2.3 to 1.9	−0.44	−2.2 to 1.4	−0.86	−2.7 to 0.94
INF-γ (pg/mL)	4.10 ± 0.43	4.00 ± 0.35	4.30 ± 0.75	4.10 ± 0.29	0.11	−0.36 to 0.59	0.18	−0.34 to 0.70	−0.15	−0.62 to 0.33	−0.082	−0.56 to 0.39
TNF-α (pg/mL)	0.64 ± 0.21	0.71 ± 0.46	0.84 ± 0.60	0.56 ± 0.55	−0.065	−0.45 to 0.32	0.28	−0.14 to 0.70	−0.2	−0.67 to 0.27	0.15	−0.32 to 0.61
**Redox biomarkers**	n = 12	n = 12	n = 11	n = 11								
iNOS (µmol/L)	20.0 ± 1.30	20.0 ± 2.80	20.0 ± 1.60	21.0 ± 1.00	0.26	−0.77 to 1.3	0.14	−0.94 to 1.2	1.1	0.069 to 2.1	0.97	−0.051 to 2.0
FRAP (µmol/L)	139.0 ± 47.00	130.0 ± 48.00	128.0 ± 32.00	130.0 ± 29.00	9.1	−16 to 34	−2	−28 to 24	12	−28 to 51	0.49	−39 to 40
TBARS (µmol/L)	4.0 ± 1.10	3.8 ± 1.50	3.0 ± 0.79	2.8 ± 0.44	−0.29	−2.1 to 1.5	−0.3	−2.2 to 1.6	−0.31	−2.1 to 1.5	−0.32	−2.1 to 1.5

Note: Data presented as mean (SD). FRAP = ferric reducing antioxidant power; iNOS = Inducible nitric oxide synthase; IL-2 = interleukin 2; IL-4 = interleukin 4; IL-6 = interleukin 6; IL-10 = interleukin 10; (E) IL-12 = interleukin 12; IFN-γ = Interferon gamma; TBARS = Thiobarbituric acid reactive substances; TNF-α = tumor necrosis factor. LFES = low frequency electrical stimulation; Pre = baseline; Post = after 10 weeks; pg/mL = Picogram/milliliter; µmol/L = Micromole/liter.

## Data Availability

Not applicable.
